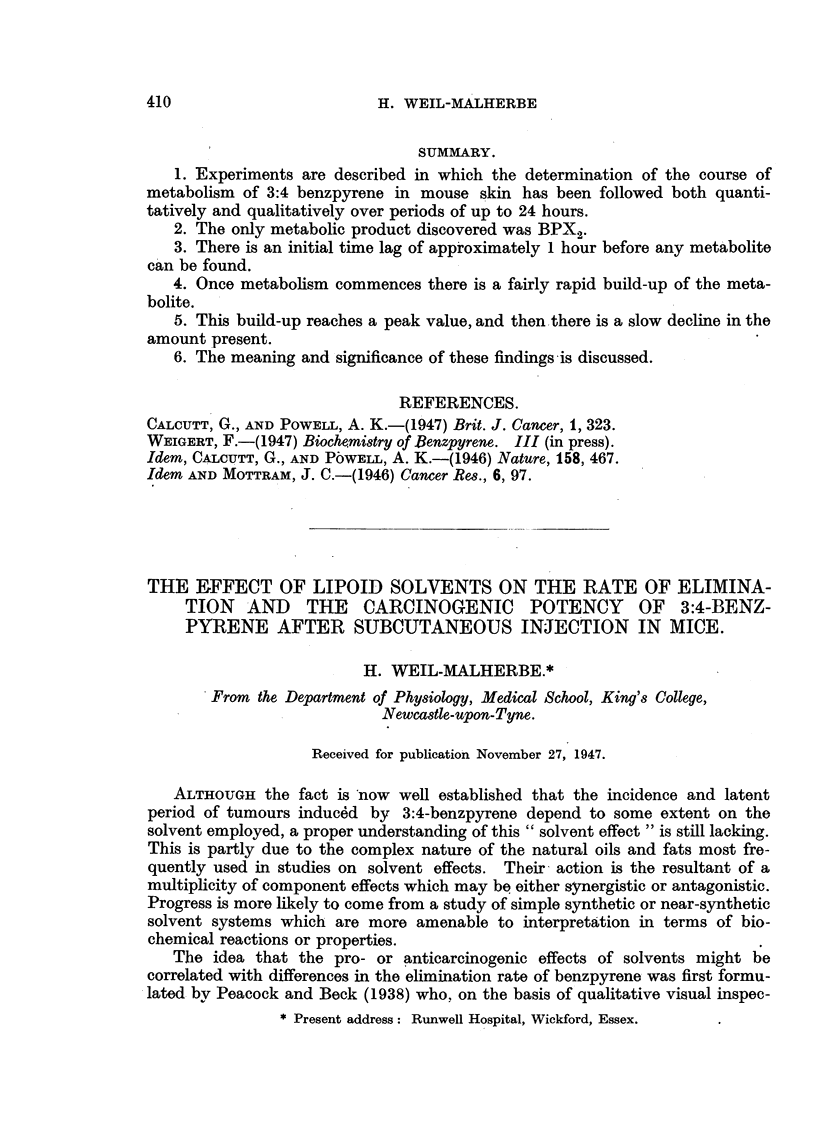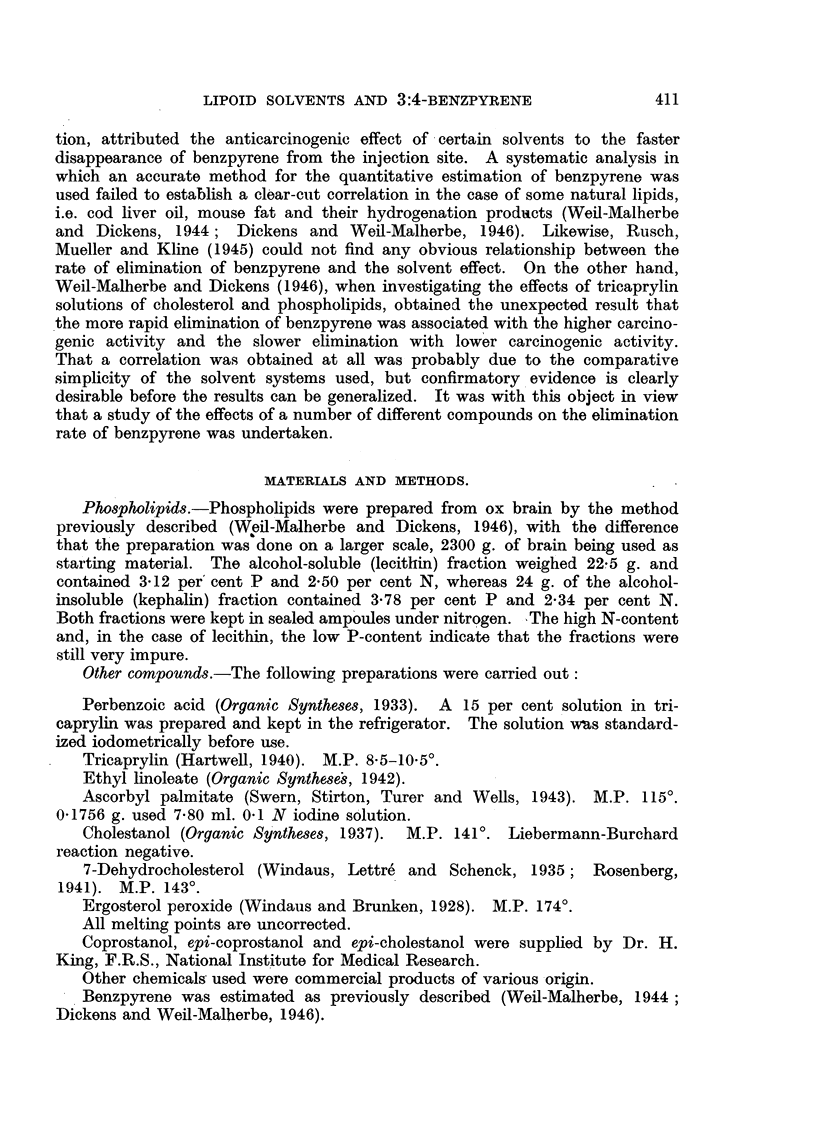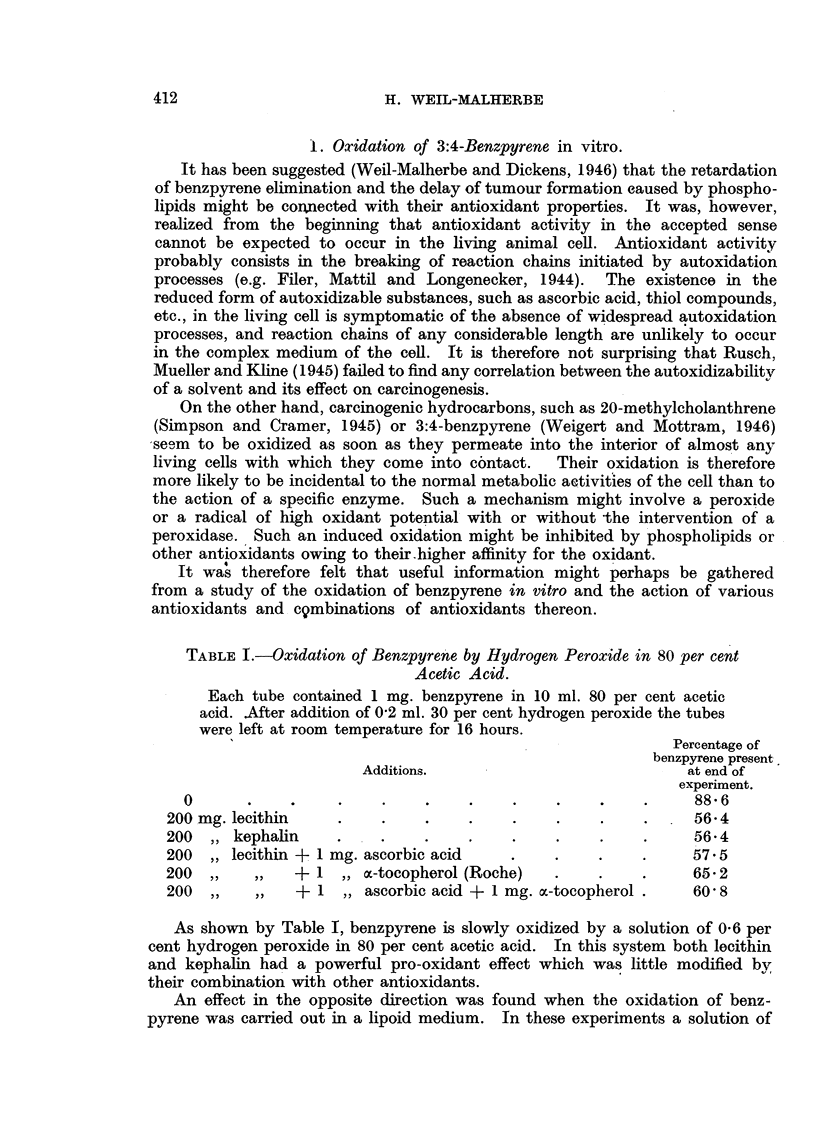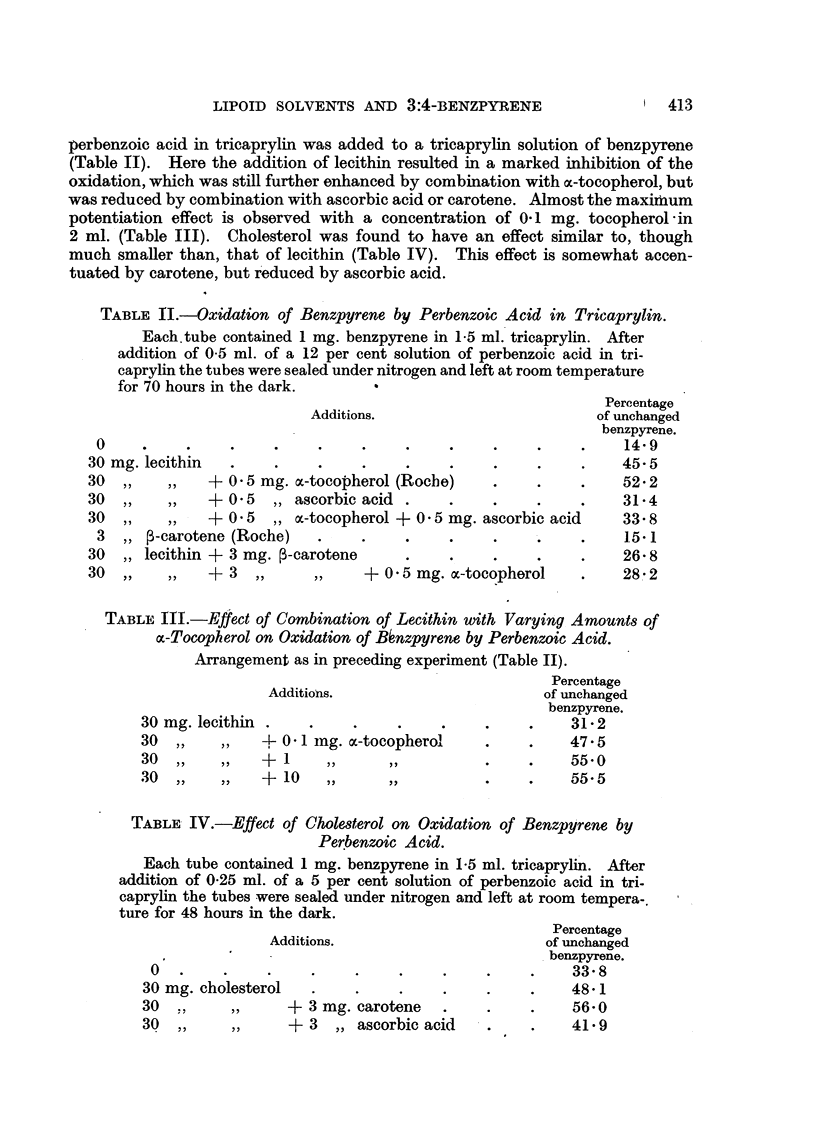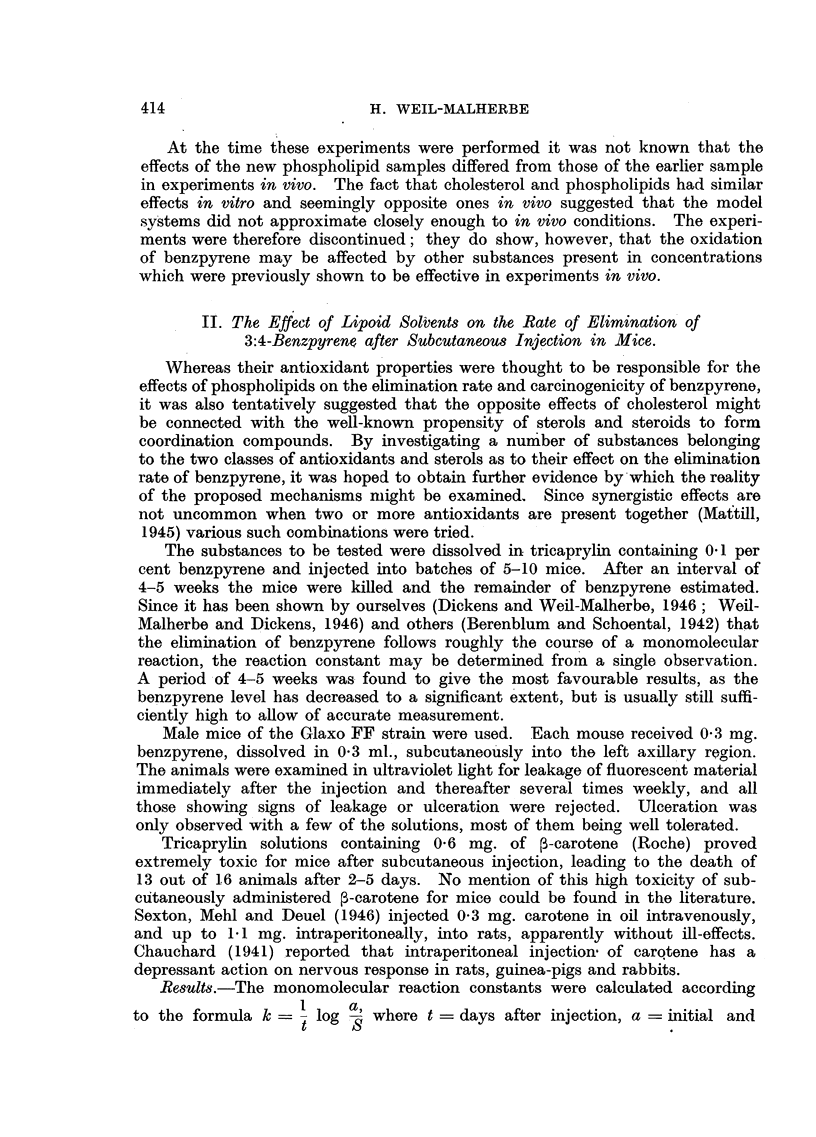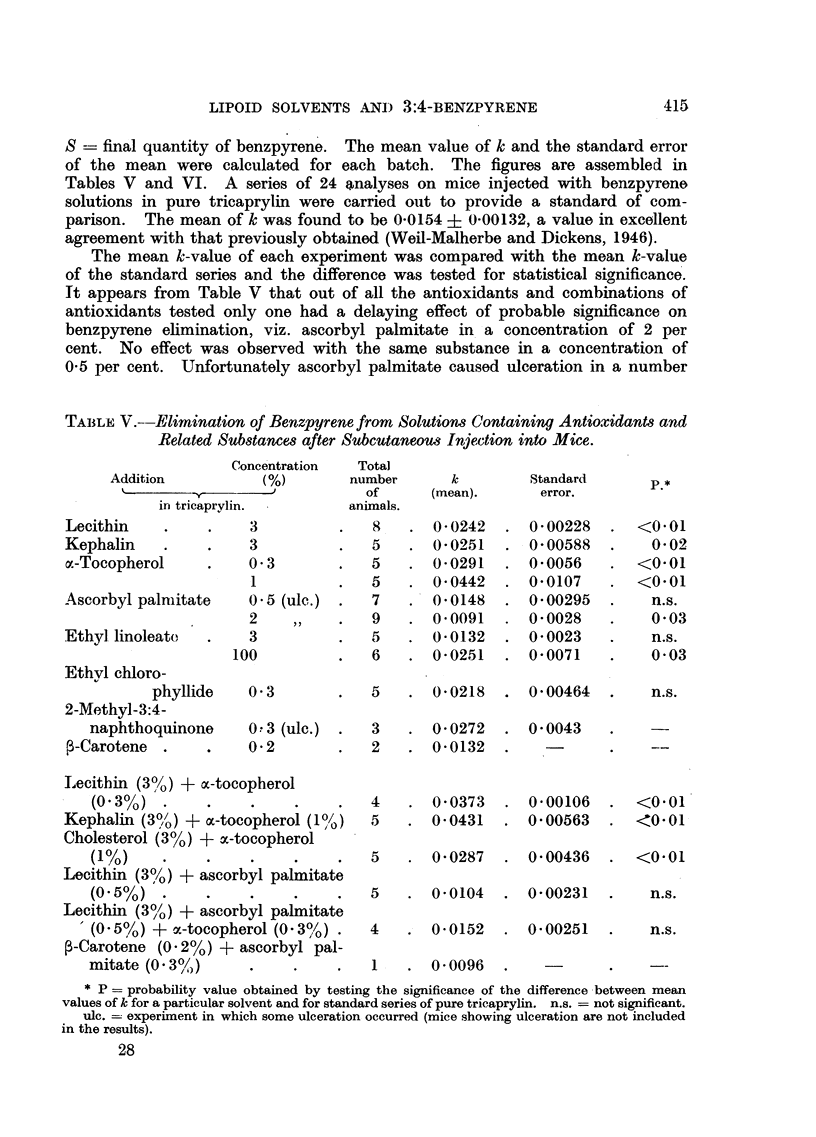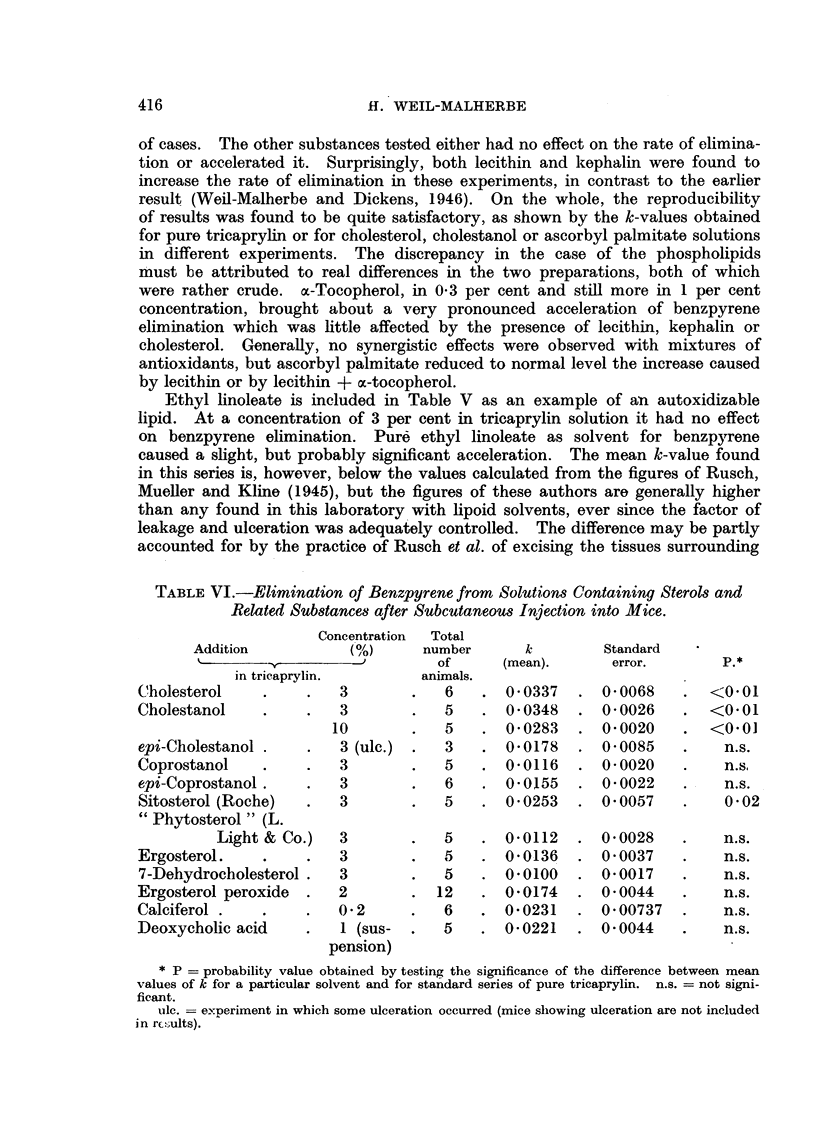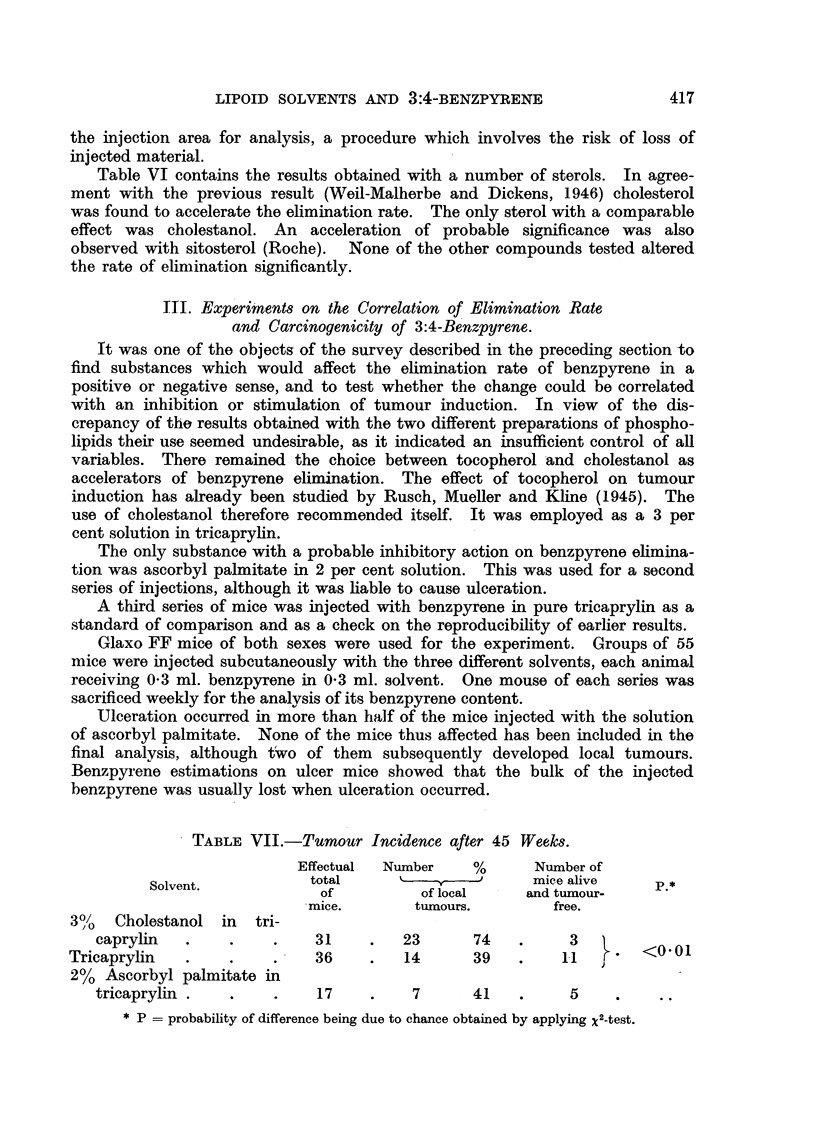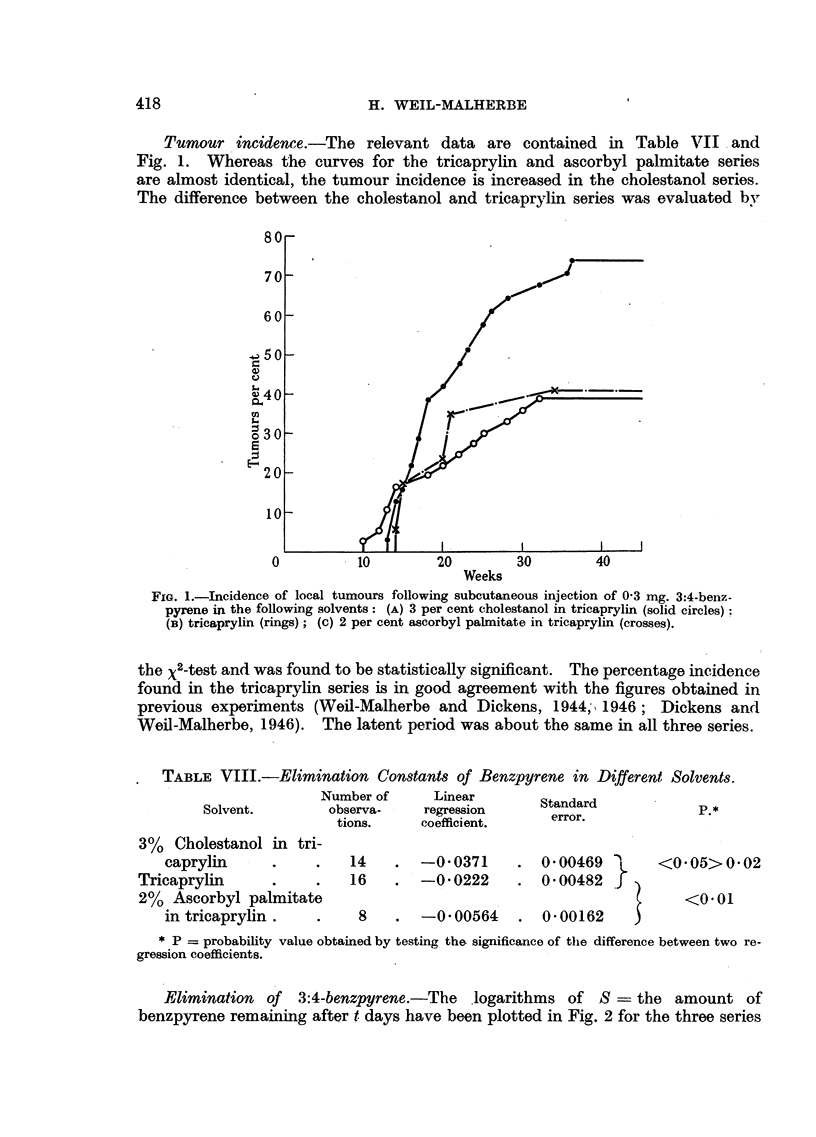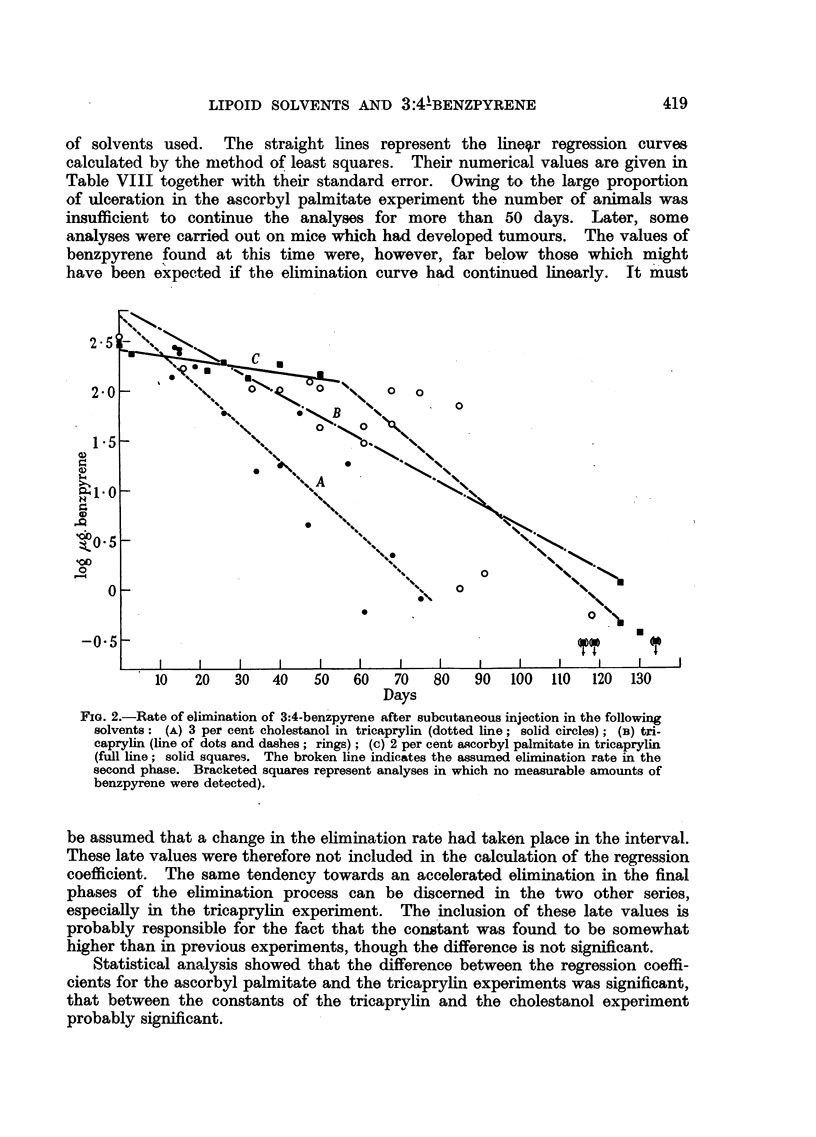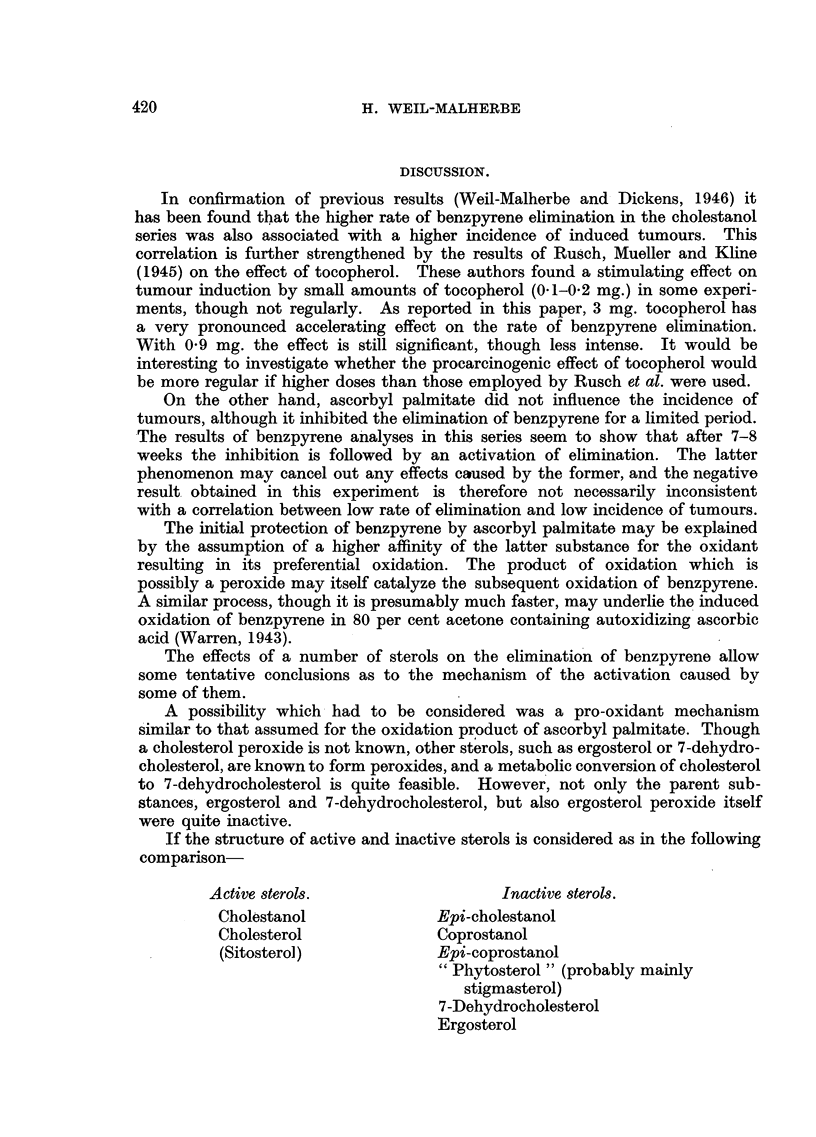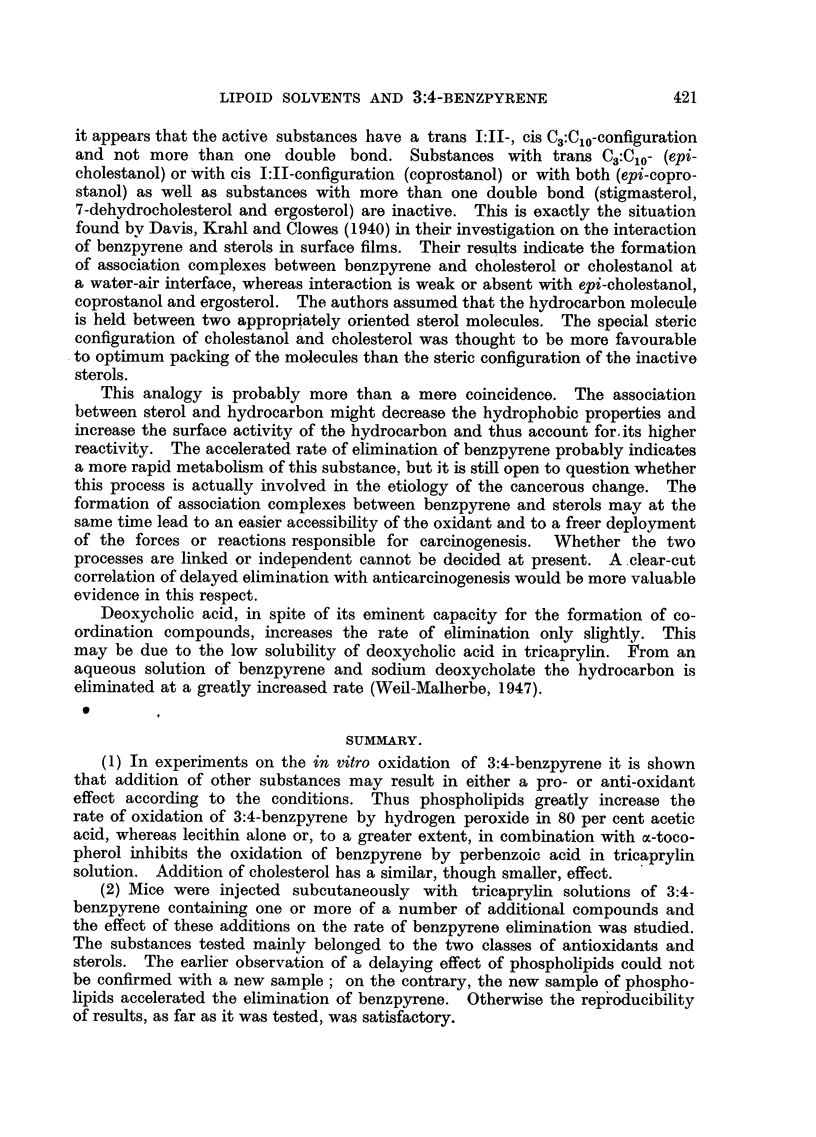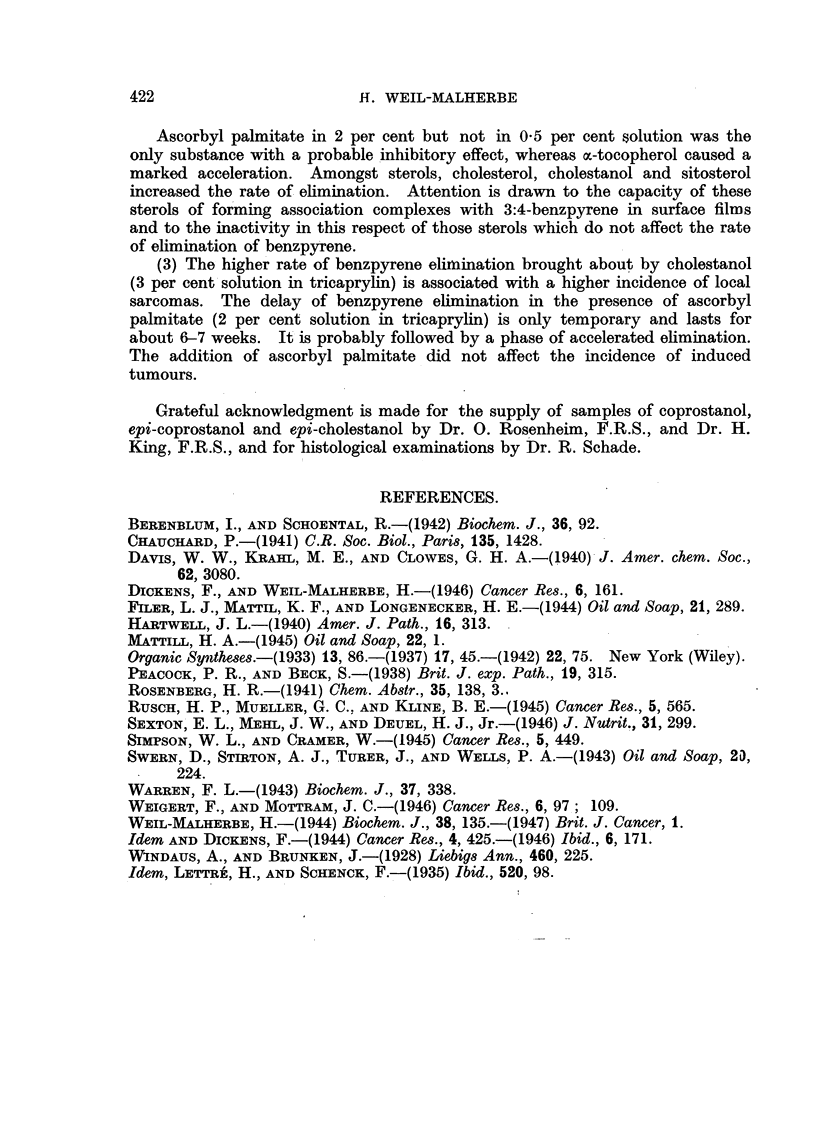# The Effect of Lipoid Solvents on the Rate of Elimination and the Carcinogenic Potency of 3:4-Benzpyrene after Subcutaneous Injection in Mice

**Published:** 1947-12

**Authors:** H. Weil-Malherbe


### THE EFFECT OF LIPOID SOLVENTS ON THE RATE OF ELIMINATION AND THE CARCINOGENIC POTENCY OF 3:4-BENZPYRENE AFTER SUBCUTANEOUS INJECTION IN MICE.

H. WEIL-MALHERBE.*

*From the Department of Physiology, Medical School, King's College, Newcastle-upon-Tyne.*

Received for publication November 27, 1947.

ALTHOUGH the fact is now well established that the incidence and latent period of tumours induced by 3:4-benzpyrene depend to some extent on the solvent employed, a proper understanding of this " solvent effect " is still lacking. This is partly due to the complex nature of the natural oils and fats most frequently used in studies on solvent effects. Their action is the resultant of a multiplicity of component effects which may be either synergistic or antagonistic. Progress is more likely to come from a study of simple synthetic or near-synthetic solvent systems which are more amenable to interpretation in terms of biochemical reactions or properties.

The idea that the pro- or anticarcinogenic effects of solvents might be correlated with differences in the elimination rate of benzpyrene was first formulated by Peacock and Beck (1938) who, on the basis of qualitative visual inspec-

* Present address : Runwell Hospital, Wickford, Essex.

tion, attributed the anticarcinogenic effect of certain solvents to the faster disappearance of benzpyrene from the injection site. A systematic analysis in which an accurate method for the quantitative estimation of benzpyrene was used failed to establish a clear-cut correlation in the case of some natural lipids, i.e. cod liver oil, mouse fat and their hydrogenation products (Weil-Malherbe and Dickens, 1944; Dickens and Weil-Malherbe, 1946). Likewise, Rusch, Mueller and Kline (1945) could not find any obvious relationship between the rate of elimination of benzpyrene and the solvent effect. On the other hand, Weil-Malherbe and Dickens (1946), when investigating the effects of tricaprylin solutions of cholesterol and phospholipids, obtained the unexpected result that the more rapid elimination of benzpyrene was associated with the higher carcinogenic activity and the slower elimination with lower carcinogenic activity. That a correlation was obtained at all was probably due to the comparative simplicity of the solvent systems used, but confirmatory evidence is clearly desirable before the results can be generalized. It was with this object in view that a study of the effects of a number of different compounds on the elimination rate of benzpyrene was undertaken.

### MATERIALS AND METHODS.

*Phospholipids.*—Phospholipids were prepared from ox brain by the method previously described (Weil-Malherbe and Dickens, 1946), with the difference that the preparation was done on a larger scale, 2300 g. of brain being used as starting material. The alcohol-soluble (lecithin) fraction weighed 22·5 g. and contained 3·12 per cent P and 2·50 per cent N, whereas 24 g. of the alcohol-insoluble (kephalin) fraction contained 3·78 per cent P and 2·34 per cent N. Both fractions were kept in sealed ampoules under nitrogen. The high N-content and, in the case of lecithin, the low P-content indicate that the fractions were still very impure.

*Other compounds.*—The following preparations were carried out:

Perbenzoic acid (*Organic Syntheses*, 1933). A 15 per cent solution in tricaprylin was prepared and kept in the refrigerator. The solution was standardized iodometrically before use.

Tricaprylin (Hartwell, 1940). M.P. 8·5–10·5°.

Ethyl linoleate (*Organic Syntheses*, 1942).

Ascorbyl palmitate (Swern, Stirton, Turer and Wells, 1943). M.P. 115°. 0·1756 g. used 7·80 ml. 0·1 *N* iodine solution.

Cholestanol (*Organic Syntheses*, 1937). M.P. 141°. Liebermann-Burchard reaction negative.

7-Dehydrocholesterol (Windaus, Lettré and Schenck, 1935; Rosenberg, 1941). M.P. 143°.

Ergosterol peroxide (Windaus and Brunken, 1928). M.P. 174°.

All melting points are uncorrected.

Coprostanol, *epi*-coprostanol and *epi*-cholestanol were supplied by Dr. H. King, F.R.S., National Institute for Medical Research.

Other chemicals used were commercial products of various origin.

Benzpyrene was estimated as previously described (Weil-Malherbe, 1944; Dickens and Weil-Malherbe, 1946).

1. *Oxidation of* 3:4-*Benzpyrene* in vitro.

It has been suggested (Weil-Malherbe and Dickens, 1946) that the retardation of benzpyrene elimination and the delay of tumour formation caused by phospholipids might be connected with their antioxidant properties. It was, however, realized from the beginning that antioxidant activity in the accepted sense cannot be expected to occur in the living animal cell. Antioxidant activity probably consists in the breaking of reaction chains initiated by autoxidation processes (e.g. Filer, Mattil and Longenecker, 1944). The existence in the reduced form of autoxidizable substances, such as ascorbic acid, thiol compounds, etc., in the living cell is symptomatic of the absence of widespread autoxidation processes, and reaction chains of any considerable length are unlikely to occur in the complex medium of the cell. It is therefore not surprising that Rusch, Mueller and Kline (1945) failed to find any correlation between the autoxidizability of a solvent and its effect on carcinogenesis.

On the other hand, carcinogenic hydrocarbons, such as 20-methylcholanthrene (Simpson and Cramer, 1945) or 3:4-benzpyrene (Weigert and Mottram, 1946) seem to be oxidized as soon as they permeate into the interior of almost any living cells with which they come into contact. Their oxidation is therefore more likely to be incidental to the normal metabolic activities of the cell than to the action of a specific enzyme. Such a mechanism might involve a peroxide or a radical of high oxidant potential with or without the intervention of a peroxidase. Such an induced oxidation might be inhibited by phospholipids or other antioxidants owing to their higher affinity for the oxidant.

It was therefore felt that useful information might perhaps be gathered from a study of the oxidation of benzpyrene *in vitro* and the action of various antioxidants and combinations of antioxidants thereon.

TABLE I.—*Oxidation of Benzpyrene by Hydrogen Peroxide in* 80 *per cent Acetic Acid.*

Each tube contained 1 mg. benzpyrene in 10 ml. 80 per cent acetic acid. After addition of 0·2 ml. 30 per cent hydrogen peroxide the tubes were left at room temperature for 16 hours.

| Additions. | Percentage of benzpyrene present at end of experiment. |
|---|---|
| 0 | 88·6 |
| 200 mg. lecithin | 56·4 |
| 200 ,, kephalin | 56·4 |
| 200 ,, lecithin + 1 mg. ascorbic acid | 57·5 |
| 200 ,, ,, + 1 ,, α-tocopherol (Roche) | 65·2 |
| 200 ,, ,, + 1 ,, ascorbic acid + 1 mg. α-tocopherol | 60·8 |

As shown by Table I, benzpyrene is slowly oxidized by a solution of 0·6 per cent hydrogen peroxide in 80 per cent acetic acid. In this system both lecithin and kephalin had a powerful pro-oxidant effect which was little modified by their combination with other antioxidants.

An effect in the opposite direction was found when the oxidation of benzpyrene was carried out in a lipoid medium. In these experiments a solution of perbenzoic acid in tricaprylin was added to a tricaprylin solution of benzpyrene (Table II). Here the addition of lecithin resulted in a marked inhibition of the oxidation, which was still further enhanced by combination with α-tocopherol, but was reduced by combination with ascorbic acid or carotene. Almost the maximum potentiation effect is observed with a concentration of 0·1 mg. tocopherol in 2 ml. (Table III). Cholesterol was found to have an effect similar to, though much smaller than, that of lecithin (Table IV). This effect is somewhat accentuated by carotene, but reduced by ascorbic acid.

TABLE II.—*Oxidation of Benzpyrene by Perbenzoic Acid in Tricaprylin.*

Each tube contained 1 mg. benzpyrene in 1·5 ml. tricaprylin. After addition of 0·5 ml. of a 12 per cent solution of perbenzoic acid in tricaprylin the tubes were sealed under nitrogen and left at room temperature for 70 hours in the dark.

| Additions. | Percentage of unchanged benzpyrene. |
|---|---|
| 0 | 14·9 |
| 30 mg. lecithin | 45·5 |
| 30 ,, ,, + 0·5 mg. α-tocopherol (Roche) | 52·2 |
| 30 ,, ,, + 0·5 ,, ascorbic acid | 31·4 |
| 30 ,, ,, + 0·5 ,, α-tocopherol + 0·5 mg. ascorbic acid | 33·8 |
| 3 ,, β-carotene (Roche) | 15·1 |
| 30 ,, lecithin + 3 mg. β-carotene | 26·8 |
| 30 ,, ,, + 3 ,, ,, + 0·5 mg. α-tocopherol | 28·2 |

TABLE III.—*Effect of Combination of Lecithin with Varying Amounts of α-Tocopherol on Oxidation of Benzpyrene by Perbenzoic Acid.*

Arrangement as in preceding experiment (Table II).

| Additions. | Percentage of unchanged benzpyrene. |
|---|---|
| 30 mg. lecithin | 31·2 |
| 30 ,, ,, + 0·1 mg. α-tocopherol | 47·5 |
| 30 ,, ,, + 1 ,, ,, | 55·0 |
| 30 ,, ,, + 10 ,, ,, | 55·5 |

TABLE IV.—*Effect of Cholesterol on Oxidation of Benzpyrene by Perbenzoic Acid.*

Each tube contained 1 mg. benzpyrene in 1·5 ml. tricaprylin. After addition of 0·25 ml. of a 5 per cent solution of perbenzoic acid in tricaprylin the tubes were sealed under nitrogen and left at room temperature for 48 hours in the dark.

| Additions. | Percentage of unchanged benzpyrene. |
|---|---|
| 0 | 33·8 |
| 30 mg. cholesterol | 48·1 |
| 30 ,, ,, + 3 mg. carotene | 56·0 |
| 30 ,, ,, + 3 ,, ascorbic acid | 41·9 |

At the time these experiments were performed it was not known that the effects of the new phospholipid samples differed from those of the earlier sample in experiments *in vivo*. The fact that cholesterol and phospholipids had similar effects *in vitro* and seemingly opposite ones *in vivo* suggested that the model systems did not approximate closely enough to *in vivo* conditions. The experiments were therefore discontinued; they do show, however, that the oxidation of benzpyrene may be affected by other substances present in concentrations which were previously shown to be effective in experiments *in vivo*.

### II. *The Effect of Lipoid Solvents on the Rate of Elimination of 3:4-Benzpyrene after Subcutaneous Injection in Mice.*

Whereas their antioxidant properties were thought to be responsible for the effects of phospholipids on the elimination rate and carcinogenicity of benzpyrene, it was also tentatively suggested that the opposite effects of cholesterol might be connected with the well-known propensity of sterols and steroids to form coordination compounds. By investigating a number of substances belonging to the two classes of antioxidants and sterols as to their effect on the elimination rate of benzpyrene, it was hoped to obtain further evidence by which the reality of the proposed mechanisms might be examined. Since synergistic effects are not uncommon when two or more antioxidants are present together (Mattill, 1945) various such combinations were tried.

The substances to be tested were dissolved in tricaprylin containing 0·1 per cent benzpyrene and injected into batches of 5–10 mice. After an interval of 4–5 weeks the mice were killed and the remainder of benzpyrene estimated. Since it has been shown by ourselves (Dickens and Weil-Malherbe, 1946; Weil-Malherbe and Dickens, 1946) and others (Berenblum and Schoental, 1942) that the elimination of benzpyrene follows roughly the course of a monomolecular reaction, the reaction constant may be determined from a single observation. A period of 4–5 weeks was found to give the most favourable results, as the benzpyrene level has decreased to a significant extent, but is usually still sufficiently high to allow of accurate measurement.

Male mice of the Glaxo FF strain were used. Each mouse received 0·3 mg. benzpyrene, dissolved in 0·3 ml., subcutaneously into the left axillary region. The animals were examined in ultraviolet light for leakage of fluorescent material immediately after the injection and thereafter several times weekly, and all those showing signs of leakage or ulceration were rejected. Ulceration was only observed with a few of the solutions, most of them being well tolerated.

Tricaprylin solutions containing 0·6 mg. of β-carotene (Roche) proved extremely toxic for mice after subcutaneous injection, leading to the death of 13 out of 16 animals after 2–5 days. No mention of this high toxicity of subcutaneously administered β-carotene for mice could be found in the literature. Sexton, Mehl and Deuel (1946) injected 0·3 mg. carotene in oil intravenously, and up to 1·1 mg. intraperitoneally, into rats, apparently without ill-effects. Chauchard (1941) reported that intraperitoneal injection of carotene has a depressant action on nervous response in rats, guinea-pigs and rabbits.

*Results.*—The monomolecular reaction constants were calculated according to the formula $k = \frac{1}{t} \log \frac{a}{S}$, where $t$ = days after injection, $a$ = initial and

$S$ = final quantity of benzpyrene. The mean value of $k$ and the standard error of the mean were calculated for each batch. The figures are assembled in Tables V and VI. A series of 24 analyses on mice injected with benzpyrene solutions in pure tricaprylin were carried out to provide a standard of comparison. The mean of $k$ was found to be $0 \cdot 0154 \pm 0 \cdot 00132$, a value in excellent agreement with that previously obtained (Weil-Malherbe and Dickens, 1946).

The mean $k$-value of each experiment was compared with the mean $k$-value of the standard series and the difference was tested for statistical significance. It appears from Table V that out of all the antioxidants and combinations of antioxidants tested only one had a delaying effect of probable significance on benzpyrene elimination, viz. ascorbyl palmitate in a concentration of 2 per cent. No effect was observed with the same substance in a concentration of 0·5 per cent. Unfortunately ascorbyl palmitate caused ulceration in a number

TABLE V.—*Elimination of Benzpyrene from Solutions Containing Antioxidants and Related Substances after Subcutaneous Injection into Mice.*

| Addition in tricaprylin. | Concentration (%) | Total number of animals. | $k$ (mean). | Standard error. | P.* |
|---|---|---|---|---|---|
| Lecithin | 3 | 8 | 0·0242 | 0·00228 | <0·01 |
| Kephalin | 3 | 5 | 0·0251 | 0·00588 | 0·02 |
| α-Tocopherol | 0·3 | 5 | 0·0291 | 0·0056 | <0·01 |
| | 1 | 5 | 0·0442 | 0·0107 | <0·01 |
| Ascorbyl palmitate | 0·5 (ulc.) | 7 | 0·0148 | 0·00295 | n.s. |
| | 2 ,, | 9 | 0·0091 | 0·0028 | 0·03 |
| Ethyl linoleate | 3 | 5 | 0·0132 | 0·0023 | n.s. |
| | 100 | 6 | 0·0251 | 0·0071 | 0·03 |
| Ethyl chlorophyllide | 0·3 | 5 | 0·0218 | 0·00464 | n.s. |
| 2-Methyl-3:4-naphthoquinone | 0·3 (ulc.) | 3 | 0·0272 | 0·0043 | — |
| β-Carotene | 0·2 | 2 | 0·0132 | — | — |
| Lecithin (3%) + α-tocopherol (0·3%) | | 4 | 0·0373 | 0·00106 | <0·01 |
| Kephalin (3%) + α-tocopherol (1%) | | 5 | 0·0431 | 0·00563 | <0·01 |
| Cholesterol (3%) + α-tocopherol (1%) | | 5 | 0·0287 | 0·00436 | <0·01 |
| Lecithin (3%) + ascorbyl palmitate (0·5%) | | 5 | 0·0104 | 0·00231 | n.s. |
| Lecithin (3%) + ascorbyl palmitate (0·5%) + α-tocopherol (0·3%) | | 4 | 0·0152 | 0·00251 | n.s. |
| β-Carotene (0·2%) + ascorbyl palmitate (0·3%) | | 1 | 0·0096 | — | — |

* P = probability value obtained by testing the significance of the difference between mean values of $k$ for a particular solvent and for standard series of pure tricaprylin. n.s. = not significant.
ulc. = experiment in which some ulceration occurred (mice showing ulceration are not included in the results).

of cases. The other substances tested either had no effect on the rate of elimination or accelerated it. Surprisingly, both lecithin and kephalin were found to increase the rate of elimination in these experiments, in contrast to the earlier result (Weil-Malherbe and Dickens, 1946). On the whole, the reproducibility of results was found to be quite satisfactory, as shown by the $k$-values obtained for pure tricaprylin or for cholesterol, cholestanol or ascorbyl palmitate solutions in different experiments. The discrepancy in the case of the phospholipids must be attributed to real differences in the two preparations, both of which were rather crude. α-Tocopherol, in 0·3 per cent and still more in 1 per cent concentration, brought about a very pronounced acceleration of benzpyrene elimination which was little affected by the presence of lecithin, kephalin or cholesterol. Generally, no synergistic effects were observed with mixtures of antioxidants, but ascorbyl palmitate reduced to normal level the increase caused by lecithin or by lecithin + α-tocopherol.

Ethyl linoleate is included in Table V as an example of an autoxidizable lipid. At a concentration of 3 per cent in tricaprylin solution it had no effect on benzpyrene elimination. Pure ethyl linoleate as solvent for benzpyrene caused a slight, but probably significant acceleration. The mean $k$-value found in this series is, however, below the values calculated from the figures of Rusch, Mueller and Kline (1945), but the figures of these authors are generally higher than any found in this laboratory with lipoid solvents, ever since the factor of leakage and ulceration was adequately controlled. The difference may be partly accounted for by the practice of Rusch *et al.* of excising the tissues surrounding

TABLE VI.—*Elimination of Benzpyrene from Solutions Containing Sterols and Related Substances after Subcutaneous Injection into Mice.*

| Addition | Concentration (%) in tricaprylin. | Total number of animals. | $k$ (mean). | Standard error. | P.* |
|---|---|---|---|---|---|
| Cholesterol | 3 | 6 | 0·0337 | 0·0068 | <0·01 |
| Cholestanol | 3 | 5 | 0·0348 | 0·0026 | <0·01 |
| | 10 | 5 | 0·0283 | 0·0020 | <0·01 |
| *epi*-Cholestanol | 3 (ulc.) | 3 | 0·0178 | 0·0085 | n.s. |
| Coprostanol | 3 | 5 | 0·0116 | 0·0020 | n.s. |
| *epi*-Coprostanol | 3 | 6 | 0·0155 | 0·0022 | n.s. |
| Sitosterol (Roche) | 3 | 5 | 0·0253 | 0·0057 | 0·02 |
| " Phytosterol " (L. Light & Co.) | 3 | 5 | 0·0112 | 0·0028 | n.s. |
| Ergosterol | 3 | 5 | 0·0136 | 0·0037 | n.s. |
| 7-Dehydrocholesterol | 3 | 5 | 0·0100 | 0·0017 | n.s. |
| Ergosterol peroxide | 2 | 12 | 0·0174 | 0·0044 | n.s. |
| Calciferol | 0·2 | 6 | 0·0231 | 0·00737 | n.s. |
| Deoxycholic acid | 1 (suspension) | 5 | 0·0221 | 0·0044 | n.s. |

* P = probability value obtained by testing the significance of the difference between mean values of $k$ for a particular solvent and for standard series of pure tricaprylin. n.s. = not significant.

ulc. = experiment in which some ulceration occurred (mice showing ulceration are not included in results).

the injection area for analysis, a procedure which involves the risk of loss of injected material.

Table VI contains the results obtained with a number of sterols. In agreement with the previous result (Weil-Malherbe and Dickens, 1946) cholesterol was found to accelerate the elimination rate. The only sterol with a comparable effect was cholestanol. An acceleration of probable significance was also observed with sitosterol (Roche). None of the other compounds tested altered the rate of elimination significantly.

### III. *Experiments on the Correlation of Elimination Rate and Carcinogenicity of* 3:4-*Benzpyrene.*

It was one of the objects of the survey described in the preceding section to find substances which would affect the elimination rate of benzpyrene in a positive or negative sense, and to test whether the change could be correlated with an inhibition or stimulation of tumour induction. In view of the discrepancy of the results obtained with the two different preparations of phospholipids their use seemed undesirable, as it indicated an insufficient control of all variables. There remained the choice between tocopherol and cholestanol as accelerators of benzpyrene elimination. The effect of tocopherol on tumour induction has already been studied by Rusch, Mueller and Kline (1945). The use of cholestanol therefore recommended itself. It was employed as a 3 per cent solution in tricaprylin.

The only substance with a probable inhibitory action on benzpyrene elimination was ascorbyl palmitate in 2 per cent solution. This was used for a second series of injections, although it was liable to cause ulceration.

A third series of mice was injected with benzpyrene in pure tricaprylin as a standard of comparison and as a check on the reproducibility of earlier results.

Glaxo FF mice of both sexes were used for the experiment. Groups of 55 mice were injected subcutaneously with the three different solvents, each animal receiving 0·3 ml. benzpyrene in 0·3 ml. solvent. One mouse of each series was sacrificed weekly for the analysis of its benzpyrene content.

Ulceration occurred in more than half of the mice injected with the solution of ascorbyl palmitate. None of the mice thus affected has been included in the final analysis, although two of them subsequently developed local tumours. Benzpyrene estimations on ulcer mice showed that the bulk of the injected benzpyrene was usually lost when ulceration occurred.

TABLE VII.—*Tumour Incidence after* 45 *Weeks.*

| Solvent. | Effectual total of mice. | Number of local tumours. | % of local tumours. | Number of mice alive and tumour-free. | P.* |
|---|---|---|---|---|---|
| 3% Cholestanol in tricaprylin | 31 | 23 | 74 | 3 | <0·01 |
| Tricaprylin | 36 | 14 | 39 | 11 | |
| 2% Ascorbyl palmitate in tricaprylin | 17 | 7 | 41 | 5 | .. |

* P = probability of difference being due to chance obtained by applying $\chi^2$-test.

*Tumour incidence.*—The relevant data are contained in Table VII and Fig. 1. Whereas the curves for the tricaprylin and ascorbyl palmitate series are almost identical, the tumour incidence is increased in the cholestanol series. The difference between the cholestanol and tricaprylin series was evaluated by

FIG. 1.—Incidence of local tumours following subcutaneous injection of 0·3 mg. 3:4-benzpyrene in the following solvents: (A) 3 per cent cholestanol in tricaprylin (solid circles); (B) tricaprylin (rings); (C) 2 per cent ascorbyl palmitate in tricaprylin (crosses).

the $\chi^2$-test and was found to be statistically significant. The percentage incidence found in the tricaprylin series is in good agreement with the figures obtained in previous experiments (Weil-Malherbe and Dickens, 1944, 1946; Dickens and Weil-Malherbe, 1946). The latent period was about the same in all three series.

TABLE VIII.—*Elimination Constants of Benzpyrene in Different Solvents.*

| Solvent. | Number of observations. | Linear regression coefficient. | Standard error. | P.* |
|---|---|---|---|---|
| 3% Cholestanol in tricaprylin | 14 | —0·0371 | 0·00469 | <0·05>0·02 (vs. Tricaprylin) |
| Tricaprylin | 16 | —0·0222 | 0·00482 | <0·01 (vs. 2% Ascorbyl palmitate) |
| 2% Ascorbyl palmitate in tricaprylin | 8 | —0·00564 | 0·00162 | |

* P = probability value obtained by testing the significance of the difference between two regression coefficients.

*Elimination of* 3:4-*benzpyrene.*—The logarithms of $S$ = the amount of benzpyrene remaining after $t$ days have been plotted in Fig. 2 for the three series of solvents used. The straight lines represent the linear regression curves calculated by the method of least squares. Their numerical values are given in Table VIII together with their standard error. Owing to the large proportion of ulceration in the ascorbyl palmitate experiment the number of animals was insufficient to continue the analyses for more than 50 days. Later, some analyses were carried out on mice which had developed tumours. The values of benzpyrene found at this time were, however, far below those which might have been expected if the elimination curve had continued linearly. It must

FIG. 2.—Rate of elimination of 3:4-benzpyrene after subcutaneous injection in the following solvents: (A) 3 per cent cholestanol in tricaprylin (dotted line; solid circles); (B) tricaprylin (line of dots and dashes; rings); (C) 2 per cent ascorbyl palmitate in tricaprylin (full line; solid squares. The broken line indicates the assumed elimination rate in the second phase. Bracketed squares represent analyses in which no measurable amounts of benzpyrene were detected).

be assumed that a change in the elimination rate had taken place in the interval. These late values were therefore not included in the calculation of the regression coefficient. The same tendency towards an accelerated elimination in the final phases of the elimination process can be discerned in the two other series, especially in the tricaprylin experiment. The inclusion of these late values is probably responsible for the fact that the constant was found to be somewhat higher than in previous experiments, though the difference is not significant.

Statistical analysis showed that the difference between the regression coefficients for the ascorbyl palmitate and the tricaprylin experiments was significant, that between the constants of the tricaprylin and the cholestanol experiment probably significant.

### DISCUSSION.

In confirmation of previous results (Weil-Malherbe and Dickens, 1946) it has been found that the higher rate of benzpyrene elimination in the cholestanol series was also associated with a higher incidence of induced tumours. This correlation is further strengthened by the results of Rusch, Mueller and Kline (1945) on the effect of tocopherol. These authors found a stimulating effect on tumour induction by small amounts of tocopherol (0·1–0·2 mg.) in some experiments, though not regularly. As reported in this paper, 3 mg. tocopherol has a very pronounced accelerating effect on the rate of benzpyrene elimination. With 0·9 mg. the effect is still significant, though less intense. It would be interesting to investigate whether the procarcinogenic effect of tocopherol would be more regular if higher doses than those employed by Rusch *et al.* were used.

On the other hand, ascorbyl palmitate did not influence the incidence of tumours, although it inhibited the elimination of benzpyrene for a limited period. The results of benzpyrene analyses in this series seem to show that after 7–8 weeks the inhibition is followed by an activation of elimination. The latter phenomenon may cancel out any effects caused by the former, and the negative result obtained in this experiment is therefore not necessarily inconsistent with a correlation between low rate of elimination and low incidence of tumours.

The initial protection of benzpyrene by ascorbyl palmitate may be explained by the assumption of a higher affinity of the latter substance for the oxidant resulting in its preferential oxidation. The product of oxidation which is possibly a peroxide may itself catalyze the subsequent oxidation of benzpyrene. A similar process, though it is presumably much faster, may underlie the induced oxidation of benzpyrene in 80 per cent acetone containing autoxidizing ascorbic acid (Warren, 1943).

The effects of a number of sterols on the elimination of benzpyrene allow some tentative conclusions as to the mechanism of the activation caused by some of them.

A possibility which had to be considered was a pro-oxidant mechanism similar to that assumed for the oxidation product of ascorbyl palmitate. Though a cholesterol peroxide is not known, other sterols, such as ergosterol or 7-dehydrocholesterol, are known to form peroxides, and a metabolic conversion of cholesterol to 7-dehydrocholesterol is quite feasible. However, not only the parent substances, ergosterol and 7-dehydrocholesterol, but also ergosterol peroxide itself were quite inactive.

If the structure of active and inactive sterols is considered as in the following comparison—

| *Active sterols.* | *Inactive sterols.* |
|---|---|
| Cholestanol | *Epi*-cholestanol |
| Cholesterol | Coprostanol |
| (Sitosterol) | *Epi*-coprostanol |
| | " Phytosterol " (probably mainly stigmasterol) |
| | 7-Dehydrocholesterol |
| | Ergosterol |

it appears that the active substances have a trans I:II-, cis $C_3$:$C_{10}$-configuration and not more than one double bond. Substances with trans $C_3$:$C_{10}$- (*epi*-cholestanol) or with cis I:II-configuration (coprostanol) or with both (*epi*-coprostanol) as well as substances with more than one double bond (stigmasterol, 7-dehydrocholesterol and ergosterol) are inactive. This is exactly the situation found by Davis, Krahl and Clowes (1940) in their investigation on the interaction of benzpyrene and sterols in surface films. Their results indicate the formation of association complexes between benzpyrene and cholesterol or cholestanol at a water-air interface, whereas interaction is weak or absent with *epi*-cholestanol, coprostanol and ergosterol. The authors assumed that the hydrocarbon molecule is held between two appropriately oriented sterol molecules. The special steric configuration of cholestanol and cholesterol was thought to be more favourable to optimum packing of the molecules than the steric configuration of the inactive sterols.

This analogy is probably more than a mere coincidence. The association between sterol and hydrocarbon might decrease the hydrophobic properties and increase the surface activity of the hydrocarbon and thus account for its higher reactivity. The accelerated rate of elimination of benzpyrene probably indicates a more rapid metabolism of this substance, but it is still open to question whether this process is actually involved in the etiology of the cancerous change. The formation of association complexes between benzpyrene and sterols may at the same time lead to an easier accessibility of the oxidant and to a freer deployment of the forces or reactions responsible for carcinogenesis. Whether the two processes are linked or independent cannot be decided at present. A clear-cut correlation of delayed elimination with anticarcinogenesis would be more valuable evidence in this respect.

Deoxycholic acid, in spite of its eminent capacity for the formation of co-ordination compounds, increases the rate of elimination only slightly. This may be due to the low solubility of deoxycholic acid in tricaprylin. From an aqueous solution of benzpyrene and sodium deoxycholate the hydrocarbon is eliminated at a greatly increased rate (Weil-Malherbe, 1947).

### SUMMARY.

(1) In experiments on the *in vitro* oxidation of 3:4-benzpyrene it is shown that addition of other substances may result in either a pro- or anti-oxidant effect according to the conditions. Thus phospholipids greatly increase the rate of oxidation of 3:4-benzpyrene by hydrogen peroxide in 80 per cent acetic acid, whereas lecithin alone or, to a greater extent, in combination with α-tocopherol inhibits the oxidation of benzpyrene by perbenzoic acid in tricaprylin solution. Addition of cholesterol has a similar, though smaller, effect.

(2) Mice were injected subcutaneously with tricaprylin solutions of 3:4-benzpyrene containing one or more of a number of additional compounds and the effect of these additions on the rate of benzpyrene elimination was studied. The substances tested mainly belonged to the two classes of antioxidants and sterols. The earlier observation of a delaying effect of phospholipids could not be confirmed with a new sample ; on the contrary, the new sample of phospholipids accelerated the elimination of benzpyrene. Otherwise the reproducibility of results, as far as it was tested, was satisfactory.

Ascorbyl palmitate in 2 per cent but not in 0·5 per cent solution was the only substance with a probable inhibitory effect, whereas α-tocopherol caused a marked acceleration. Amongst sterols, cholesterol, cholestanol and sitosterol increased the rate of elimination. Attention is drawn to the capacity of these sterols of forming association complexes with 3:4-benzpyrene in surface films and to the inactivity in this respect of those sterols which do not affect the rate of elimination of benzpyrene.

(3) The higher rate of benzpyrene elimination brought about by cholestanol (3 per cent solution in tricaprylin) is associated with a higher incidence of local sarcomas. The delay of benzpyrene elimination in the presence of ascorbyl palmitate (2 per cent solution in tricaprylin) is only temporary and lasts for about 6–7 weeks. It is probably followed by a phase of accelerated elimination. The addition of ascorbyl palmitate did not affect the incidence of induced tumours.

Grateful acknowledgment is made for the supply of samples of coprostanol, *epi*-coprostanol and *epi*-cholestanol by Dr. O. Rosenheim, F.R.S., and Dr. H. King, F.R.S., and for histological examinations by Dr. R. Schade.

### REFERENCES.

Berenblum, I., and Schoental, R.—(1942) *Biochem. J.*, **36**, 92.
Chauchard, P.—(1941) *C.R. Soc. Biol., Paris*, **135**, 1428.
Davis, W. W., Krahl, M. E., and Clowes, G. H. A.—(1940) *J. Amer. chem. Soc.*, **62**, 3080.
Dickens, F., and Weil-Malherbe, H.—(1946) *Cancer Res.*, **6**, 161.
Filer, L. J., Mattil, K. F., and Longenecker, H. E.—(1944) *Oil and Soap*, **21**, 289.
Hartwell, J. L.—(1940) *Amer. J. Path.*, **16**, 313.
Mattill, H. A.—(1945) *Oil and Soap*, **22**, 1.
*Organic Syntheses*.—(1933) **13**, 86.—(1937) **17**, 45.—(1942) **22**, 75. New York (Wiley).
Peacock, P. R., and Beck, S.—(1938) *Brit. J. exp. Path.*, **19**, 315.
Rosenberg, H. R.—(1941) *Chem. Abstr.*, **35**, 138, 3..
Rusch, H. P., Mueller, G. C., and Kline, B. E.—(1945) *Cancer Res.*, **5**, 565.
Sexton, E. L., Mehl, J. W., and Deuel, H. J., Jr.—(1946) *J. Nutrit.*, **31**, 299.
Simpson, W. L., and Cramer, W.—(1945) *Cancer Res.*, **5**, 449.
Swern, D., Stirton, A. J., Turer, J., and Wells, P. A.—(1943) *Oil and Soap*, **20**, 224.
Warren, F. L.—(1943) *Biochem. J.*, **37**, 338.
Weigert, F., and Mottram, J. C.—(1946) *Cancer Res.*, **6**, 97 ; 109.
Weil-Malherbe, H.—(1944) *Biochem. J.*, **38**, 135.—(1947) *Brit. J. Cancer*, **1**.
*Idem* and Dickens, F.—(1944) *Cancer Res.*, **4**, 425.—(1946) *Ibid.*, **6**, 171.
Windaus, A., and Brunken, J.—(1928) *Liebigs Ann.*, **460**, 225.
*Idem*, Lettré, H., and Schenck, F.—(1935) *Ibid.*, **520**, 98.